# Identification and Functional Validation of Key Regulatory Genes for Leaf Development in Moso Bamboo (*Phyllostachys edulis*) Based on Transcriptome Data

**DOI:** 10.3390/plants15111673

**Published:** 2026-05-29

**Authors:** Zhuo Lv, Na Wang, Simei Ai, Hanjiao Zhang, Shuyan Lin

**Affiliations:** 1 State Key Laboratory for Development and Utilization of Forest Food Resources, Co-Innovation Center for Sustainable Forestry in Southern China, Bamboo Research Institute, Nanjing 210037, China; zlv@njfu.edu.cn (Z.L.);; 2 College of Life Sciences, Nanjing Forestry University, Nanjing 210037, China; 3 College of Forestry and Grassland, Nanjing Forestry University, Nanjing 210037, China

**Keywords:** leaf development, molecular mechanism, morphogenesis, Moso bamboo, transcriptome sequencing

## Abstract

To systematically elucidate the molecular regulatory mechanisms underlying leaf development in Moso bamboo, this study conducted transcriptome sequencing and systematic analysis on leaves across three key developmental stages: the division stage, the elongation stage, and the mature stage. A total of 25,256 differentially expressed genes (DEGs) were identified. Functional enrichment analysis revealed the sequential molecular events during leaf development: pathways related to cell division and hormone signal transduction were highly active in the early developmental stages, whereas photosynthesis and secondary metabolism were significantly enriched in the mature stage. This reflects the dynamic transition of leaves from morphogenesis to physiological function. Further analysis screened out 3390 transcription factors, among which the AP2/EREBP and bHLH families exhibited notably dynamic expression patterns. Subsequent transgenic functional validation demonstrated that the overexpression of *PheANT* led to an increased number of leaves and morphological abnormalities in *Arabidopsis*, while the overexpression of *PhebHLH137* resulted in slender leaves and early flowering. This study systematically maps the transcriptomic landscape of Moso bamboo leaf development and identifies multiple key regulatory genes, providing important theoretical insights and candidate gene resources for revealing the unique leaf development mechanisms in bamboo plants.

## 1. Introduction

Moso bamboo is an important perennial woody bamboo species belonging to the Bambusoideae subfamily of the Poaceae family. It is characterized by rapid growth and fast biomass accumulation. As the core organ for photosynthesis and morphogenesis, the leaf development mechanism directly affects the overall growth efficiency of the plant [1]. An in-depth understanding of the molecular basis of Moso bamboo leaf development will help reveal the intrinsic mechanisms underlying the rapid growth of bamboo species and provide theoretical support for genetic improvement. Plant leaf development is a precisely regulated biological process involving the coordination of multiple stages, such as cell division, expansion, and differentiation [2]. In model plants such as *Arabidopsis thaliana* and rice, key regulatory pathways, including hormone signal transduction, have been gradually elucidated [3,4,5]. The development of high-throughput transcriptome sequencing technology provides an effective means to systematically reveal the gene expression profiles during dynamic organ development in plants. This technology is also applicable to non-model plants like Moso bamboo, for which genetic transformation systems are not yet well established.

Transcription factors play a central role in regulating plant development. Among them, the AP2/EREBP and bHLH families are two important transcription factor families widely involved in leaf morphogenesis. Members of the AP2/EREBP family affect leaf shape and size by regulating cell division, differentiation, hormone signaling, and miRNA networks. For example, mutation of an *AP2* gene in maize leads to leaf curling [6]; mutation of the rice *OsDRB2* gene reduces miR166 accumulation and upregulates *HD-ZIP III* expression, resulting in inward leaf rolling [7]. The bHLH family plays key roles in leaf architecture establishment and stomatal differentiation. For instance, rice *OsbHLH98* negatively regulates leaf angle by modulating the number and size of adaxial parenchyma cells in the lamina joint [8]; the *Arabidopsis* SPEECHLESS transcription factor initiates stomatal lineage cell differentiation and is a key regulator of stomatal formation in the leaf epidermis [9]. These findings indicate that the AP2/EREBP and bHLH transcription factor families have conserved and diversified regulatory functions in leaf morphogenesis across various plant species.

Although the regulatory functions of the AP2/EREBP and bHLH transcription factor families in leaf morphogenesis have been widely confirmed in model plants, the dynamic changes in the transcriptome during Moso bamboo leaf development, the key genes and signaling pathways governing morphogenesis at different stages, and the specific regulatory mechanisms of the two families in this process remain unclear. Therefore, this study focuses on three key stages of Moso bamboo leaf development, namely the division stage, elongation stage, and maturation stage. We systematically analyze the dynamic transcriptome changes and gene expression patterns associated with morphogenesis. The aim is to identify the key genes and signaling pathways that dominate leaf morphogenesis at each stage and to further reveal the regulatory mechanisms of AP2/EREBP and bHLH family genes in Moso bamboo leaf morphogenesis. Through the identification and functional validation of key genes, this study expects to provide a systematic understanding of the molecular regulatory network underlying Moso bamboo leaf development, thereby laying a theoretical foundation for elucidating the rapid growth mechanisms of bamboo species and for their genetic improvement.

## 2. Materials and Methods

### 2.1. Moso Bamboo Seedling Materials and Growth Conditions

**Figure 1:**
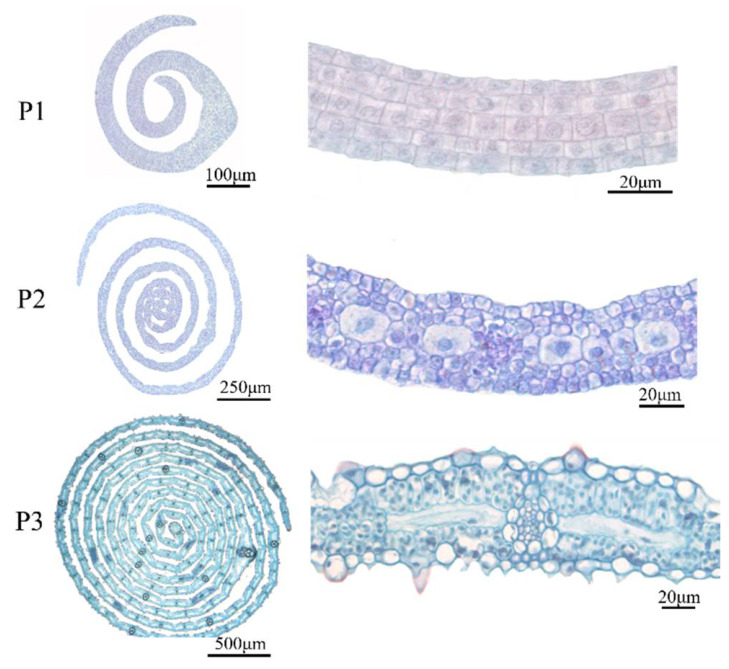
Transverse section structure of Moso bamboo leaves at three different developmental stages. P1: division stage; P2: elongation stage; P3: maturation stage.

The leaf morphology of Moso bamboo (*Phyllostachys edulis*) seedlings is significantly larger than that of adult forest plants, and its genome data are publicly available. Therefore, this study selected seedling leaves as materials for leaf development research. The experimental materials used in this study were 4-year-old Moso bamboo seedlings, planted at the Beida Mountain Laboratory of Nanjing Forestry University (geographical coordinates: 118°49′11″ E, 32°04′48″ N; altitude 8.9 m). This region has a subtropical monsoon climate, with an annual average temperature of 15.7 °C, an annual precipitation of 1106.5 mm, and uneven seasonal rainfall distribution (spring 23%, summer 45%, autumn 21%); the annual average relative humidity is 76%. Based on the cytological characterization of leaf morphogenesis in Moso bamboo, leaves at the division stage, elongation stage, and maturation stage were selected for transcriptome sequencing, and these three stages were designated as P1, P2, and P3 (Figure 1), respectively. After collection, the samples were immediately frozen in liquid nitrogen and stored. Each sample had three biological replicates.

### 2.2. Total RNA Extraction, Library Construction, and Transcriptome Sequencing

Total RNA was extracted from the samples using the TIANGEN RNAprep Pure Plant Kit (TIANGEN Biotech (Beijing, China). Cat. No. DP441) following the manufacturer’s instructions. RNA integrity and potential DNA contamination were assessed using agarose gel electrophoresis. RNA purity (OD_260/280_ and OD_260/230_ ratios) was measured with a NanoPhotometer spectrophotometer (Implen GmbH, Munich, Germany). RNA concentration was precisely quantified using a Qubit 2.0 Fluorometer (Invitrogen, Carlsbad, CA, USA), and RNA integrity was further evaluated using an Agilent 2100 Bioanalyzer (Agilent Technologies, Santa Clara, CA, USA). Subsequently, strand-specific RNA-seq libraries were constructed and sequenced according to the Illumina standard protocol (Genedenovo, Guangzhou, China).

### 2.3. Transcriptome Data Processing, Assembly, and Expression Quantification

Fastp [10] (v0.18.0) was used for quality control of the raw RNA-Seq reads. Reads containing adapters, reads with a proportion of N bases greater than 10%, reads consisting entirely of A bases, and low-quality reads (where the proportion of bases with a quality value Q ≤ 20 exceeded 50% of the entire read) were filtered out to obtain clean reads. Subsequently, Bowtie2 [11] (version 2.2.8) was employed to precisely align the quality-controlled clean reads to the transcriptome. Based on the alignment results, HISAT2 [12] was then used with default parameters to align the reads to the second version of the Moso bamboo reference genome (https://gigadb.org/dataset/100498, accessed on 12 August 2024) [13]. On this basis, using the above alignment results, StringTie [14,15] (v1.3.1) was applied to assemble transcripts for each sample in a reference genome-dependent manner, and RSEM [16] was further used to calculate the FPKM value for each transcribed region.

### 2.4. Differential Expression Analysis and Functional Enrichment Analysis

For gene expression analysis, FPKM normalization was applied, and the filtering criteria were FDR < 0.05 and |log2FC| > 2. The identified DEGs were mapped to the Gene Ontology (GO) and Kyoto Encyclopedia of Genes and Genomes (KEGG) databases for functional annotation. Cytoscape (version 3.10.2) [17] was employed to construct gene interaction networks for the important genes screened.

### 2.5. Experimental Materials for Genetic Transformation of Arabidopsis

(1) Vectors and strains: The overexpression vector used for the target genes was pCAMBIA1302. *Escherichia coli* DH5α competent cells and *Agrobacterium tumefaciens* GV3101 competent cells were purchased from Sangon (Shanghai, China).
(2) PCR primer synthesis and sequencing: Gene-specific primers and plasmid DNA sequencing were commissioned to Tsingke (Nanjing, China).
(3) Plant material for genetic transformation: *Arabidopsis thaliana* (L.) Heynh. ecotype *Columbia* (*Col-0*) was used for genetic transformation experiments. The growth substrate for *Arabidopsis* was prepared at a ratio of peat moss: vermiculite: perlite = 9:3:1. The growth conditions were 24 °C with a photoperiod of 16 h light/8 h dark.

### 2.6. Gene Cloning and Construction of Overexpression Vector for Arabidopsis

(1) RNA extraction and cDNA synthesis: Total RNA was extracted using the FastPure Universal Plant Total RNA Isolation Kit (Vazyme, Nanjing, China) following the manufacturer’s instructions. Moso bamboo cDNA was synthesized using the TransScript One-Step gDNA Removal and cDNA Synthesis Super Mix (TransGen Biotech, AT311, Beijing, China) according to the manufacturer’s protocol.
(2) Target fragment amplification and gel extraction: Primers were designed using Primer Premier 5 (Table S1). Using the cDNA from Moso bamboo leaves as a template, the target fragment was amplified by PCR with 2× Phanta UniFi Master Mix (Dye Plus) (Nanjing, China) high-fidelity enzyme and corresponding specific primers. The PCR products were analyzed by 1% agarose gel electrophoresis, and the target gene PCR products were purified using the FastPure Gel DNA Extraction Mini Kit (Vazyme, DC301, Nanjing, China). The collected DNA products were stored at –20 °C for subsequent experiments.
(3) Cloning vector construction: The pCAMBIA1302 vector was linearized by double digestion with *Nco*I-HF and *BstE*II-HF restriction enzymes (New England Biolabs, Ipswich, MA, USA). The linearized vector was then subjected to homologous recombination using the ClonExpress Multi One Step Cloning Kit V3 (Vazyme, CI15, Nanjing, China) to construct the target gene expression vector.
(4) *E. coli* transformation: Five microliters of the recombination product were added to 50 μL of *E. coli* DH5α competent cells. The mixture was gently tapped to mix, incubated on ice for 30 min, heat-shocked in a 42 °C water bath for 30 s, and then placed on ice for another 3 min. Subsequently, 900 μL of antibiotic-free LB liquid medium was added, and the cells were recovered by shaking at 37 °C, 200 rpm for 60 min. After centrifugation at 5000 rpm for 3 min, 700 μL of supernatant was discarded. The cell pellet was gently resuspended in the remaining supernatant using a pipette. One hundred microliters of the suspension were spread onto LB solid medium plates containing kanamycin (50 μg/mL) and incubated upside down at 37 °C for no more than 16 h.
(5) Positive clone verification: Single white colonies with smooth, intact margins were picked and thoroughly mixed in 10 μL of ddH_2_O. One microliter of the bacterial suspension was used as a PCR template for amplification with pCAMBIA1302-specific primers and 2× Rapid Taq Master Mix (Vazyme, P222, Nanjing, China). The PCR products were analyzed by 1% agarose gel electrophoresis. Positive clones showing the correct target band size were selected, inoculated into 350 μL of LB liquid medium containing kanamycin (50 μg/mL), and shaken at 37 °C, 200 rpm for 3 h. The bacterial cultures were then sent to Tsingke (Nanjing, China) for sequencing verification. The sequencing results were aligned with the target gene nucleotide sequences, and only those with perfect matches were used for subsequent experiments.

### 2.7. Genetic Transformation of Arabidopsis

(1) *A. tumefaciens* transformation: Five microliters of the sequencing-verified target gene plasmid were added to *A. tumefaciens* GV3101 competent cells. The mixture was incubated on ice for 5 min, then in liquid nitrogen for 5 min, followed by a 5 min water bath at 37 °C, and finally an additional 5 min on ice. Subsequently, 700 μL of antibiotic-free YEB liquid medium were added, and the cells were cultured at 28 °C with shaking at 200 rpm for 3 h. After centrifugation at 6000 rpm for 3 min, 500 μL of supernatant were discarded, and the cell pellet was gently resuspended. One hundred microliters of the bacterial suspension were spread onto LB solid medium plates containing kanamycin (50 μg/mL) and rifampicin (20 μg/mL), and incubated upside down at 28 °C for 2–3 days.
(2) Positive clone verification: The experimental procedure was the same as described in Section 2.6 (5).
(3) *A. tumefaciens*-mediated transformation of *Arabidopsis*: Single colonies of *A. tumefaciens* harboring the target gene were picked and inoculated into YEB liquid medium containing kanamycin and rifampicin. The culture was shaken until OD_600_ reached 0.8–1.0, then centrifuged at 4500 rpm for 10 min. The supernatant was discarded, and the bacterial pellet was collected and resuspended in a suspension solution composed of sucrose and a surfactant to serve as the infiltration buffer. The flower buds of *Arabidopsis* were immersed in the *A. tumefaciens* infiltration buffer for 30 s, then the plant surface was blotted dry, and a second infiltration was performed for another 30 s. After infiltration, the plants were placed in the dark for 24 h and then transferred to the plant growth room for normal cultivation. One week after the first infiltration, a second infiltration was performed during the full flowering stage to improve transformation efficiency. Once the *Arabidopsis* plants matured, T_0_ seeds were harvested in bulk for each gene, dried at 28 °C, and stored in silica gel desiccators for subsequent identification.

### 2.8. Screening and Molecular Identification of Transgenic Positive Lines

(1) Screening of positive *Arabidopsis* seedlings: Seeds from T_0_ transgenic and Wild-Type (WT) *Arabidopsis* lines were surface-sterilized with 75% ethanol. The seeds in a centrifuge tube were gently shaken for 5–7 min. The 75% ethanol was removed, and 1 mL of absolute ethanol was added. The seeds were then pipetted onto sterilized filter paper and allowed to dry completely. The sterilized WT seeds were evenly sown on antibiotic-free 1/2 MS solid medium for WT plant cultivation. The transgenic seeds were evenly sown on 1/2 MS solid medium containing hygromycin (25 mg/L), stratified at 4 °C for 2 days, and then transferred to a light incubator (conditions: 24 °C, 16 h light/8 h dark). After 10 days of culture, healthy seedlings with four true leaves were selected and transplanted into mixed substrate, covered with plastic wrap, and thoroughly watered. After one day, the plastic wrap was removed and normal light culture was resumed.
(2) Identification of positive seedlings by RT-PCR: When the transgenic *Arabidopsis* plants reached the fourth week (before bolting), leaves from both transgenic and WT plants were collected for DNA extraction using the RoomTemp Sample Lysis Kit (Vazyme, P073, Nanjing, China). One microliter of the lysed DNA solution was used as a template for PCR amplification with Vazyme 2× Rapid Taq Master Mix (Nanjing, China). All PCR products were analyzed by 1% agarose gel electrophoresis, and those showing the correct band size were preliminarily identified as positive seedlings.
(3) Identification of positive seedlings by qRT-PCR: Total RNA was extracted from four-week-old transgenic *Arabidopsis* leaves, and cDNA was synthesized using the TransScript All-in-One First-Strand cDNA Synthesis Kit (TransGen, AT341, Beijing, China) according to the manufacturer’s instructions. *At-actin* was used as the internal reference gene. Primers for qRT-PCR of candidate genes and the reference gene were designed using Primer Premier 5 software (Table S1). Using the reverse-transcribed cDNA as template, qRT-PCR was performed with the ChamQ Universal SYBR qPCR Master Mix (Vazyme, Nanjing, China). After amplification, the relative expression levels of candidate genes were calculated using the 2^−ΔCt^ method relative to the control.

### 2.9. Subcellular Localization

The ORF sequence of the target gene was fused with the GFP green fluorescent reporter protein and cloned into the expression vector pCAMBIA1302. Primer information for the target gene is provided in Table S1. Transient transformation was performed in *Nicotiana benthamiana* leaves, with RFP (red fluorescent protein) used as a co-localization control. The transient transformation of tobacco leaves was commissioned to BioRun (Wuhan, China).

### 2.10. Data Analysis and Figure Preparation

Graphs were generated using GraphPad Prism 8 software, and figure layouts were prepared in Adobe Illustrator 2024. Leaf chlorophyll and nitrogen contents were measured using a plant nutrient meter TYS-A (Hangzhou, China). The measurement ranges were 0.0–99.9 SPAD for chlorophyll and 0.0–99.9 mg/g for nitrogen content, with repeatability errors of ±0.3 SPAD for chlorophyll (within the SPAD range of 0–50) and ±0.5 units for nitrogen content.

## 3. Results

### 3.1. Transcriptomic Characteristics and Identification of Differential Genes During Leaf Development in Moso Bamboo

#### 3.1.1. Overview of Illumina Transcriptome Sequencing Data During Leaf Development

Quality control analysis was performed on the nine samples from this Illumina RNA sequencing, and the results showed that all samples exhibited good sequencing quality (Table S2). In terms of raw data, each sample contained abundant raw reads, ranging from 42,580,302 to 48,373,984, providing a sufficient data foundation for subsequent analyses. After filtering, the proportion of clean data in all samples was consistently high, ranging from 99.30% to 99.72%, indicating an extremely high percentage of effective data. The total number of reads per sample corresponded to the clean data volume, ranging from 42,332,282 to 48,002,538, further confirming the adequacy of effective data. Regarding sequence alignment efficiency, the percentage of total mapped reads in each sample was excellent, ranging from 94.84% to 96.12%, demonstrating high matching specificity between the sequencing reads and the reference genome, and accurately reflecting the transcriptomic information of the samples. Collectively, these quality control metrics indicate that the Illumina RNA sequencing data are of high quality, with a high proportion of clean data and ideal alignment rates, thereby satisfying the requirements for subsequent transcriptomic analyses.

#### 3.1.2. Sample Relationships and Differential Analysis During Leaf Development

**Figure 2:**
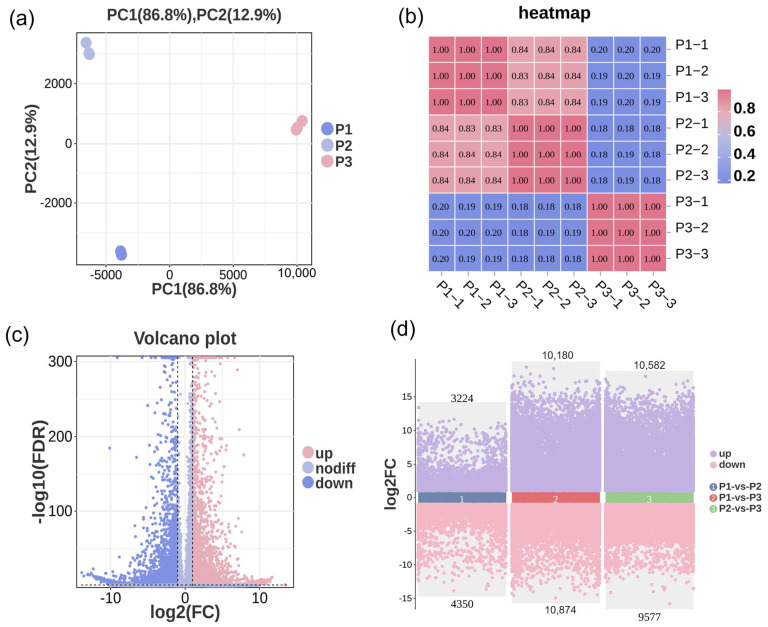
Analysis of transcriptome sequencing data and differentially expressed genes across different sample groups. (**a**) Principal component analysis (PCA) showing the overall transcriptomic differences and sample reproducibility among three biological replicate groups; (**b**) Heatmap of Pearson correlation coefficients between samples, used to evaluate the similarity of gene expression levels across different sample groups. P1, P2, and P3 represent three stages of leaf development: P1 (division stage), P2 (elongation stage), and P3 (maturation stage); (**c**) Volcano plot of differentially expressed genes. The *x*-axis represents the log_2_ fold change (log_2_(FC)) in gene expression, and the *y*-axis represents the negative logarithm of the false discovery rate (−log_10_(FDR)). Pink dots indicate significantly upregulated genes (up), dark blue dots indicate significantly downregulated genes (down), and light blue dots indicate genes with no significant difference (nodiff); (**d**) Scatter plot of differential expression for each comparison group. Blue dots represent significantly upregulated genes, and pink dots represent significantly downregulated genes.

**Figure 3:**
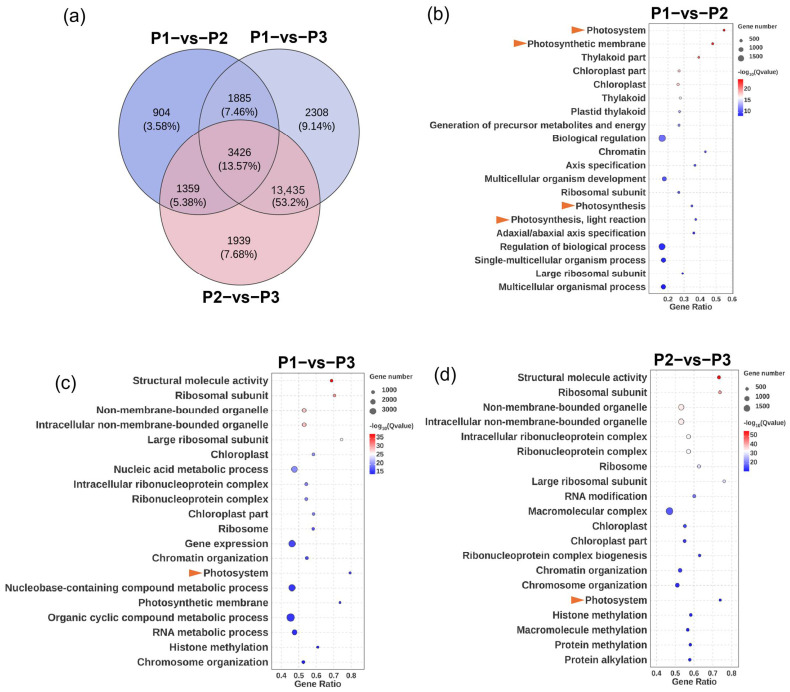
Differential expression analysis and Gene Ontology (GO) functional enrichment analysis among different comparison groups. (**a**) Venn diagram of differentially expressed genes in each comparison group. (**b**) Bubble chart of GO functional enrichment analysis for differentially expressed genes in the P1 vs. P2 group. Bubble size represents the number of enriched genes, and color represents the significance level (−log_10_(Q-value)). (**c**) Bubble chart of GO functional enrichment analysis for differentially expressed genes in the P1 vs. P3 group. (**d**) Bubble chart of GO functional enrichment analysis for differentially expressed genes in the P2 vs. P3 group. Orange triangles indicate significantly enriched pathways.

Principal component analysis (PCA) was performed on the RNA-seq data of three groups of Moso bamboo leaves at different developmental stages. As shown in Figure 2a, the PCA results based on FPKM values revealed a clear separation of sample distributions among P1, P2, and P3 at different developmental stages. Correlation analysis among samples showed that the three biological replicates of the same sample had a very high correlation (correlation coefficient of 1.0). Samples from P1 and P2 also exhibited a relatively high correlation (0.84), whereas correlations among P1, P2, and P3 samples were lower (Figure 2b). Moreover, there are a large number of statistically significant upregulated and downregulated genes with large fold changes between leaves of different developmental stages of Moso bamboo (Figure 2c). By screening the transcriptome data from different leaf developmental stages (FDR < 0.05, |log2FC| > 2), a total of 25,256 differentially expressed genes (DEGs) were identified (Figure 2d). In the comparison between the division stage and the elongation stage (P1/P2), there were 7574 DEGs, of which 3224 were upregulated and 4350 were downregulated. In the comparison between the division stage and the maturation stage (P1/P3), 21,054 DEGs were identified, including 10,180 upregulated and 10,874 downregulated. In the comparison between the elongation stage and the maturation stage (P2/P3), 20,159 DEGs were identified, with 10,582 upregulated and 9577 downregulated. Analysis of the overlapping DEGs among the different comparison groups revealed 3426 common DEGs across all three comparisons. Notably, 13,435 common DEGs were shared between P1/P3 and P2/P3, representing the largest proportion, accounting for 53.2% of all DEGs (Figure 3a).

### 3.2. Molecular Regulatory Network Dissection of Leaf Development

#### 3.2.1. Differential Gene Enrichment Analysis During Leaf Development of Moso Bamboo

Gene Ontology (GO) enrichment analysis was performed on DEGs from different comparison groups (Figure S1). During leaf development, a total of 53 GO terms were enriched, including 24 Biological Process (BP) terms, 12 Molecular Function (MF) terms, and 17 Cellular Component (CC) terms. BP terms were mainly enriched in metabolic process, cellular process, and single-organism process; MF terms were mainly enriched in catalytic activity and binding; CC terms were mainly enriched in cell, cell part, and organelle (Figure S1). In the P1/P2 comparison group (Figure 3b), CC terms were significantly enriched in photosystem, photosynthetic membrane, and thylakoid part, indicating that mesophyll cells gradually began to form at the P2 stage. BP terms were mainly enriched in generation of precursor metabolites and energy, and biological regulation. MF terms were mainly enriched in structural molecule activity and tetrapyrrole binding. In the P1/P3 and P2/P3 comparison groups (Figure 3c,d), CC terms were mainly enriched in ribosomal subunit and non-membrane-bounded organelle; MF terms were mainly enriched in structural molecule activity and nucleic acid binding. For BP terms, the P1/P3 comparison group was mainly enriched in nucleic acid metabolic process and gene expression, while the P2/P3 comparison group was mainly enriched in RNA modification and ribonucleoprotein complex biogenesis. The GO enrichment results at different developmental stages indicate that biosynthetic and nucleic acid-related functions undergo certain changes as leaves develop.

**Figure 4:**
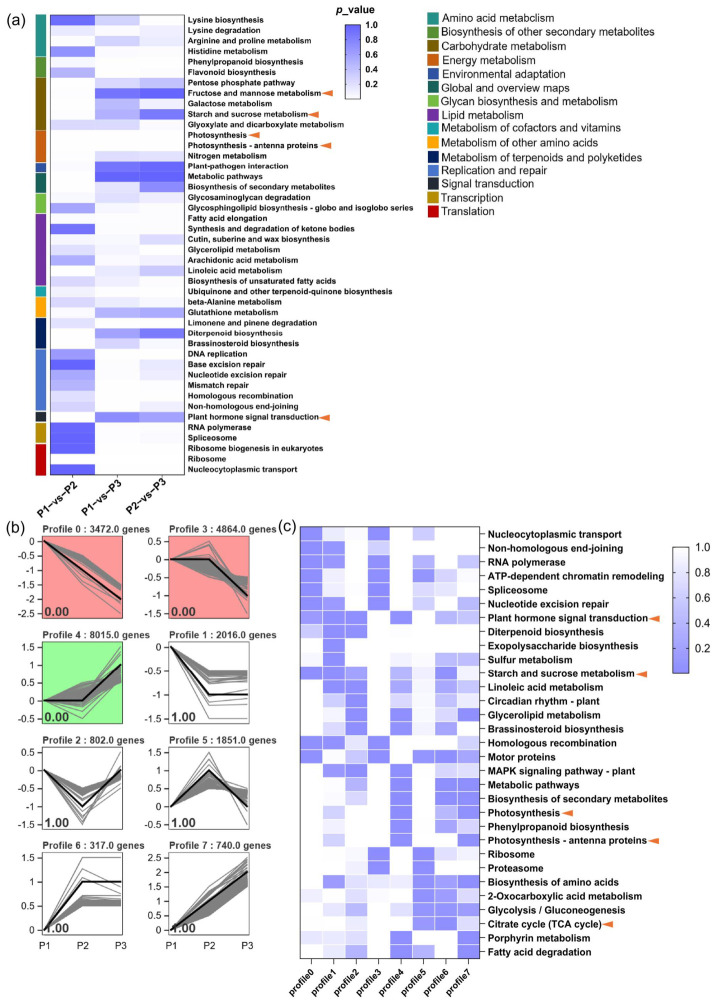
KEGG pathway enrichment, expression trend clustering, and functional characterization analysis of differentially expressed genes. (**a**) Heatmap of KEGG pathway enrichment for differentially expressed genes (DEGs) in the three comparison groups. The colored bars on the left indicate the metabolic categories to which the pathways belong (e.g., amino acid metabolism, carbohydrate metabolism, energy metabolism). Color intensity represents the enrichment significance (*p*-value), with darker colors indicating higher *p*-values and less significant enrichment. (**b**) Results of gene expression pattern clustering analysis, showing 8 significant expression profiles (Profile 0–7). Red and green represent significantly different profiles, with red indicating significant down-regulation and green indicating significant up-regulation. In each subplot, gray lines represent the expression trajectory of individual genes, and the thick black line represents the average trend of the profile. The *x*-axis represents the sample groups (P1, P2, P3), and the *y*-axis represents the normalized expression level. The subplot title indicates the number of genes in the profile, and the value in the bottom right corner represents the profile significance (*p*-value). (**c**) Heatmap of KEGG pathway enrichment for each expression profile (Profile 0–7). Color intensity represents the enrichment significance. The *y*-axis indicates the names of enriched KEGG pathways, and the *x*-axis represents the expression profile categories. Orange triangles indicate significantly enriched pathways.

KEGG enrichment analysis was performed on the DEGs during Moso bamboo leaf development (Figure 4a). At the early stage of leaf development (P1/P2), the DEGs were mainly enriched in pathways such as the pentose phosphate pathway, fructose and mannose metabolism, galactose metabolism, starch and sucrose metabolism, and plant hormone signal transduction. Sugar metabolism provides raw materials for cell wall synthesis and energy during leaf development, supporting cell division and expansion, while also facilitating the initiation and maintenance of photosynthesis and coordinating the transition of leaves from sink to source. Meanwhile, plant hormones synergistically regulate cell division, differentiation, expansion, and functional specialization through complex signaling networks. As leaf development progressed, the DEGs were mainly enriched in pathways such as photosynthesis, photosynthesis-antenna proteins, flavonoid biosynthesis, and cutin, suberin and wax biosynthesis. The enrichment of flavonoid biosynthesis indicates that leaves begin to accumulate defensive secondary metabolites, thereby enhancing stress resistance.

#### 3.2.2. Trend Analysis of Differential Genes During Leaf Development of Moso Bamboo

Trend analysis was performed on the differentially expressed genes, generating a total of eight trend modules (Figure 4b). Among these eight modules, three significantly different modules–Profile 0, Profile 3, and Profile 4–were identified. Profile 3 contained 4864 DEGs, exhibiting an expression pattern that remained stable during the division and elongation stages but decreased at the maturation stage. Profile 4 contained 8015 DEGs, showing an expression pattern that remained stable during the division and elongation stages but increased at the maturation stage. Profile 0 contained 3472 DEGs, with an expression trend that decreased progressively as leaves developed. Based on KEGG enrichment analysis (Figure 4c), the modules displayed functional differentiation and coordinated regulation during Moso bamboo leaf development. Profile 0 and Profile 3 were mainly enriched in pathways such as nucleocytoplasmic transport, plant hormone signal transduction, and starch and sucrose metabolism, which regulate cell division and elongation. Profile 4 was mainly enriched in pathways such as photosynthesis, photosynthesis-antenna proteins, flavonoid biosynthesis, MAPK signaling pathway (plant), phenylpropanoid biosynthesis, and brassinosteroid (BR) biosynthesis.

#### 3.2.3. Dynamic Regulation of Key Metabolic Pathways

**Figure 5:**
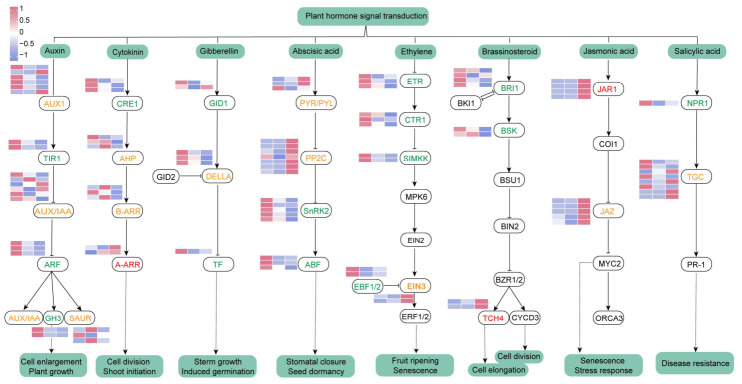
Plant hormone signal transduction pathways and expression patterns of key genes. Note: In the heatmap, color intensity represents the expression level of genes in the corresponding pathway, with red indicating upregulation and blue indicating downregulation. Each heatmap position corresponds to a specific gene. The color of the gene name indicates the regulation type: red for upregulation, green for downregulation, and orange for genes that show both upregulation and downregulation. One gene name corresponds to multiple IDs. The T–shaped arrow (⊥) represents negative regulation, and the arrow (→) represents positive regulation.

During Moso bamboo leaf development, the plant hormone signal transduction pathway was significantly enriched in different comparison groups, playing a dominant role. As shown in Figure 4, 97 DEGs were significantly expressed during leaf development. Among them, genes in the auxin and cytokinin (CK) pathways were activated at the early developmental stage and functioned throughout the entire process from initiation to maturation, regulating cell division and expansion at the early stage. In the auxin pathway (Figure 5), *TIR1*, *ARF*, and *GH3* were downregulated, synergistically supporting organ primordium initiation, regulating cell proliferation, and establishing polarity at the early stage, while *AUX1*, *AUX1/IAA*, and *SAUR* showed both upregulation and downregulation during development. In the cytokinin pathway, *CRE1* was downregulated but highly expressed at the early stage, promoting division and proliferation of leaf primordium cells, participating in leaf primordium initiation and early morphogenesis, and maintaining cell proliferation potential. *A-ARR* was upregulated, negatively feedback-inhibiting cytokinin signaling, terminating cell division to promote differentiation and maturation, maintaining functional homeostasis to ensure efficient physiological activities, and coordinating temporal transition to prepare for orderly senescence. In the gibberellin (GA) pathway, *GID1* and *TF* were highly expressed at the early stage, responding to GA signals, driving proliferation and elongation of leaf primordium cells, participating in leaf polarity establishment and photosynthetic function initiation, and providing molecular drive for the transition from leaf primordium to mature leaf. In the abscisic acid (ABA) pathway, *SnRK2* and *ABF* were highly expressed at the early stage and downregulated as leaves developed; SnRK2 regulated cell proliferation at the early stage, and ABF regulated polarity establishment, differentiation threshold, and defense gene expression. In the ethylene pathway, *ETR*, *CTR1*, *SIMKK*, and *EBF1/2* were highly expressed at the early stage, reinforcing the negative regulatory network of the ethylene pathway, suppressing premature activation of downstream response genes, and ensuring orderly progression of early core events such as cell proliferation and polarity establishment. As leaves developed, their expression decreased, releasing inhibition, allowing ethylene signaling to moderately participate in leaf maturation, functional differentiation, and environmental adaptation. In the jasmonic acid (JA) pathway, *JAR1* was upregulated during leaf development, regulating defense of mature leaves against external stress and coordinating senescence-associated gene expression and nutrient recycling. In the salicylic acid (SA) pathway, *NPR1* was highly expressed at the early stage, enhancing CK signaling activity to regulate cell proliferation, inhibiting excessive activation of the SA pathway, and forming an antagonistic balance with the JA/ethylene pathway.

**Figure 6:**
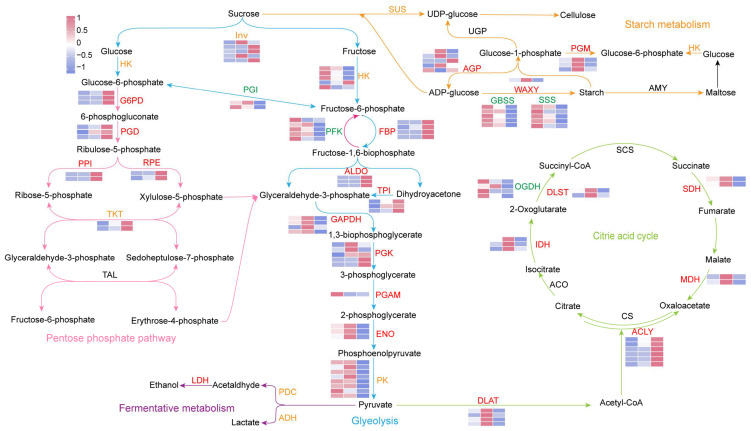
Heatmap showing the expression patterns of key enzyme-coding genes in carbon metabolism-related pathways. Note: In the heatmap, color intensity represents the expression level of genes in the corresponding pathway, with red indicating upregulation and blue indicating downregulation. Each heatmap position corresponds to a specific gene. The color of the gene name indicates the regulation type: red for upregulation, green for downregulation, and orange for genes that show both upregulation and downregulation. One gene name corresponds to multiple IDs. Arrows indicate the generation relationships among metabolites.

Carbohydrate metabolism plays an important role during Moso bamboo leaf development. The starch and sucrose metabolism pathway is a core branch of leaf carbohydrate metabolism. Sugars, as the central energy source, drive cell proliferation and structural construction. Through synthesis, degradation, conversion, and transport, these processes support the entire leaf life cycle from juvenility to maturation and senescence. During Moso bamboo leaf development, the expression of ADP-glucose pyrophosphorylase (AGP) and phosphoglucomutase (PGM) was upregulated (Figure 6), promoting substantial starch synthesis in mature leaves. In contrast, granule-bound starch synthase (GBSS) and soluble starch synthase (SSS) were downregulated, facilitating starch degradation and reducing energy consumption. In addition, other metabolic pathways such as the tricarboxylic acid (TCA) cycle, glycolysis, and the pentose phosphate pathway (PPP) also played important roles. Among these, α-ketoglutarate dehydrogenase (OGDH) in the TCA cycle, as well as phosphoglucose isomerase (PGI) and phosphofructokinase (PFK) in the glycolysis pathway, were downregulated. At the early stage of leaf development, cell division and volume expansion require extensive carbohydrate breakdown to provide energy and synthetic precursors. High expression of PGI accelerates the conversion of glucose-6-phosphate (G-6-P) to fructose-6-phosphate (F-6-P), rapidly generating the glycolytic intermediate F-6-P and supplying sufficient substrate for PFK-catalyzed reactions. PFK is highly expressed at the early stage, providing energy for cell division and expansion, thereby ensuring rapid proliferation of young cells. During the transition of Moso bamboo leaves from primordium to maturity, the overall trend of carbohydrate metabolism pathways is upregulated. At the early stage, enhanced glycolysis, the TCA cycle, and the PPP accelerate ATP generation and material synthesis, providing raw materials (such as UDP-glucose and amino acid precursors) for cell division, cell wall construction, and organelle maturation. After leaves mature, increased efficiency of the TCA cycle and sucrose/starch metabolism promotes photosynthetic energy production and product allocation, supporting self-metabolism and energy supply to sink organs.

**Figure 7:**
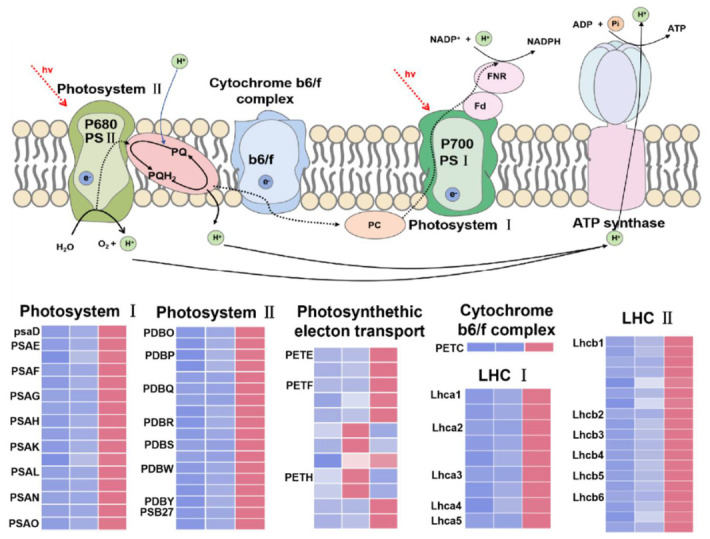
Schematic diagram of the electron transport chain during the light reaction of photosynthesis and heatmap of key gene expression. Note: The upper panel illustrates the electron transport process involving PSII, Cytochrome b6/f complex, PSI, and ATP synthase. The lower panel shows the expression heatmaps of key genes associated with Photosystem I, Photosystem II, photosynthetic electron transport, Cytochrome b6/f complex, and light-harvesting chlorophyll-protein complexes (LHC I, LHC II). Color intensity represents changes in gene expression levels, with red indicating upregulation and blue indicating downregulation. Arrows represent the pathways and directions of electron transfer, proton transport, and energy metabolism in the light reaction.

During Moso bamboo leaf development, the dynamic expression of photosynthesis-related genes is crucial for the establishment of photosynthetic function. A total of 75 photosynthesis-related DEGs were identified in this study (Figure 7). Among them, 26 genes encoding photosynthetic antenna proteins were significantly upregulated, including the photosystem I (PSI) antenna protein Lhca and the photosystem II (PSII) antenna protein Lhcb. These antenna proteins are key components for light capture; their high expression expands the light absorption range and enhances light capture efficiency, providing sufficient energy for the light reactions during leaf development. Core component genes of both PSI and PSII showed an upregulation trend, promoting the conversion of light energy into chemical energy. Meanwhile, one DEG encoding a component of the cytochrome b6/f complex was upregulated. This complex serves as a key hub connecting the two photosystems, and its enhanced expression accelerates electron transfer efficiency and coordinates the functional coupling between PSI and PSII. Furthermore, the overall upregulation of photosynthetic electron transport-related genes ensures efficient operation of the electron transport chain, promotes the generation of ATP and NADPH, and supplies sufficient energy for carbon assimilation in the dark reactions.

### 3.3. Transcriptional Regulation of Leaf Morphogenesis

#### 3.3.1. Screening of Core Transcription Factor Families

**Figure 8:**
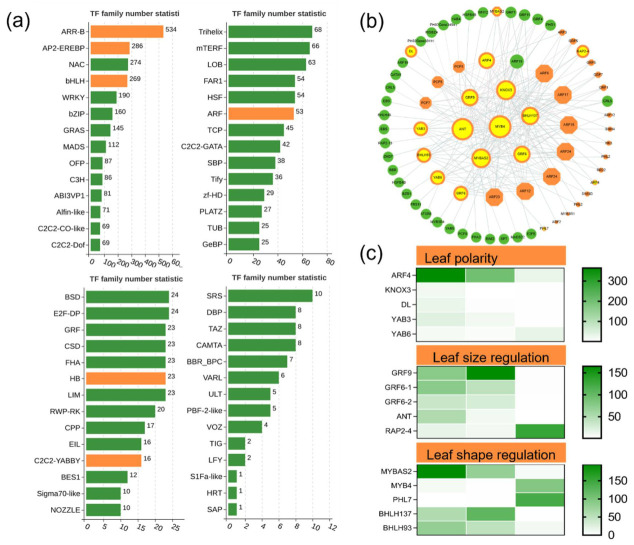
Analysis of transcription factor regulation during the development of Moso bamboo leaves. (**a**) TFs annotated during the development of Moso bamboo leaves. Orange represents the transcription factor family of interest related to leaf morphogenesis. (**b**) Transcription factor regulatory network. Circle size represents the number of interacting proteins; larger circles indicate more core transcription factors. Line density represents interaction density; nodes with more connections are more critical in the network. Yellow highlighting represents core transcription factors. (**c**) Key regulatory transcription factors. Color indicates expression level; darker color means higher expression.

During Moso bamboo leaf development, a total of 3390 genes were annotated into 56 transcription factor families (Figure 8a). Among them, the most abundant families were ARR-B, AP2-EREBP, ANC, and bHLH, containing 534, 286, 274, and 269 genes, respectively. Based on the functions of transcription factor families, 15 core transcription factors from six families related to leaf development were screened (Figure 8b,c). Among these, MYBAS2, MYB4, and PHL7 from the ARR-B family [18,19], and BHLH137 and BHLH93 from the bHLH family regulate leaf shape [20]. ARF4 from the ARF family, KNOX3 from the HB family [21], and DL [22], YAB3, and YAB6 from the C2C2-YABBY family regulate leaf polarity [23]. GRF9 and GRF6 from the GRF family [24], as well as ANT and RAP2-4 from the AP2-EREBP family, regulate leaf size [25]. These 15 core transcription factors all play important roles in leaf development. However, due to the workload associated with experimental validation, it is difficult to verify their functions one by one. Taking into account expression levels, fold change, and network centrality, this study selected PheANT and PhebHLH137 as representative transcription factors for subsequent validation. The remaining core transcription factors also hold significant research value and await further in-depth investigation.

#### 3.3.2. Functional Validation of Key Genes

##### Phenotypic Analysis of Transgenic Arabidopsis Overexpressing *PheANT*, a Leaf Shape Regulatory Gene

**Figure 9:**
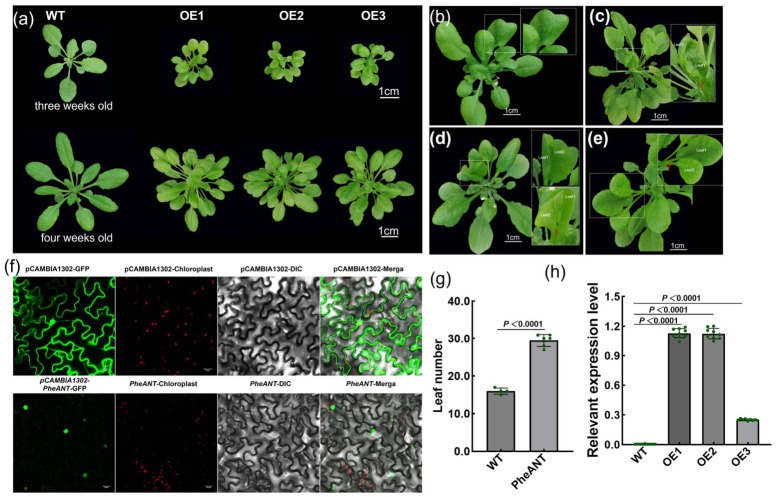
Phenotypic analysis of *PheANT* transgenic *Arabidopsis*. (**a**) Phenotypes of *PheANT* transgenic *Arabidopsis* and the control at three and four weeks of age. (**b**–**e**) Leaf phenotypic characteristics of *PheANT* transgenic *Arabidopsis*, (**b**) heart-shaped leaves with a bifurcated main vein, (**c**) dorsiventrally symmetrical paired leaves, (**d**,**e**) asymmetric paired leaves at the apex of the petiole. The leaves indicated by the red arrows are those with abnormal morphology. (**f**) Subcellular localization. (**g**) Number of leaves. (**h**) Expression levels of *PheANT* in transgenic lines.

The AP2/EREBP (APETALA2/Ethylene-Responsive Element Binding Protein) transcription factor family plays multifaceted regulatory roles during plant leaf development. This family regulates both leaf shape and size throughout leaf development. Phenotypic observation of *PheANT*-overexpressing *Arabidopsis* plants revealed an increased leaf number phenotype, as shown in Figure 9a,g. Upon overexpression of *PheANT* in *Arabidopsis*, the transgenic plants exhibited distinct leaf morphological abnormalities, which could be classified into three main patterns. The first pattern was a heart-shaped leaf with a bifurcated main vein, characterized by disordered venation resulting in two main veins radiating divergently from the petiole tip toward the leaf margin. The overall leaf shape was broad and heart-like, markedly different from the single-veined lanceolate leaves of the wild type (Figure 9b). The second pattern was dorsoventrally symmetrical double leaves: at a single petiole tip, two leaves differentiated symmetrically along the dorsoventral axis (the adaxial–abaxial axis) and were arranged toward the abaxial side (Figure 9c). The third pattern was asymmetrical double leaves at the petiole tip, where a single petiole produced two asymmetrical leaves (Figure 9d,e); this was the most frequently observed abnormal pattern. These phenotypic features suggest that *PheANT* overexpression significantly affects leaf number and venation development in *Arabidopsis*. Subcellular localization results indicated that *PheANT* predominantly functions in the nucleus (Figure 9f). qRT-PCR analysis showed that the relative expression level of *PheANT* in leaf tissues of transgenic plants was elevated compared to that in WT controls (Figure 9h). Taken together, these results demonstrate that *PheANT* regulates leaf number and morphology by modulating cell proliferation and expansion in leaves.

##### Phenotypic Analysis of Transgenic Arabidopsis Overexpressing *PhebHLH137*, a Leaf Shape Regulatory Gene

**Figure 10:**
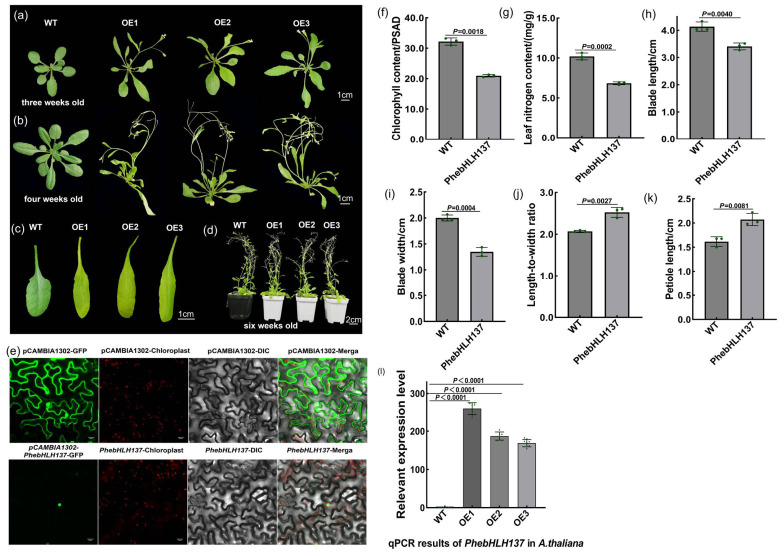
Phenotypic analysis of *PhebHLH137* transgenic *Arabidopsis*. (**a**,**b**) Phenotypes of *PhebHLH137* transgenic *Arabidopsis* leaves and controls at three and four weeks of age; (**c**) Phenotype of a single leaf at four weeks of age; (**d**) Phenotypes of six-week-old *Arabidopsis*; (**e**) Results of subcellular localization; (**f**,**g**) Relative chlorophyll content (**f**) and nitrogen content (**g**) in leaves of *PhebHLH137* overexpressing *Arabidopsis*; (**h**–**k**) Changes in leaf length (**h**), width (**i**), length-to-width ratio (**j**), and petiole length (**k**) during the growth of *PhebHLH137* transgenic *Arabidopsis* plants and controls; (**l**) Expression levels in *PhebHLH137* transgenic lines.

To elucidate the regulatory function of the *PhebHLH137* gene on leaf shape in Moso bamboo, an overexpression vector of *PhebHLH137* was constructed and introduced into the model plant *Arabidopsis* via *A. tumefaciens*-mediated genetic transformation. Stable transgenic lines overexpressing *PhebHLH137* were obtained. Comparative phenotypic observation of the transgenic lines and WT *Arabidopsis* revealed significant differences. As shown in Figure 10, overexpression of *PhebHLH137* in *Arabidopsis* led to a pronounced early-flowering phenotype, with bolting and flowering beginning at the third week. The rosette leaves were slender and elongated, and the leaf color was yellower than that of the wild type (Figure 10a–d). Further measurements (Figure 10h,i) showed that at the fourth week after transplantation, the transgenic plants exhibited significant changes in rosette leaf length, width, length-to-width ratio, and petiole length. Specifically, leaf width decreased, the length-to-width ratio increased, and petiole length increased, collectively resulting in an elongated leaf morphology. As shown in Figure 10f,g, the relative chlorophyll content and nitrogen content in rosette leaves of *PhebHLH137*-overexpressing transgenic plants were significantly reduced, indicating that overexpression of this gene affects photosynthesis and nutrient metabolism. Subcellular localization results (Figure 10e) showed that *PhebHLH137* predominantly functions in the nucleus. qRT-PCR analysis revealed that the relative expression level of *PhebHLH137* in leaf tissues of transgenic plants was elevated compared to that in WT (Figure 10l). These results indicate that overexpression of *PhebHLH137* not only affects the growth and development of *Arabidopsis* but also significantly interferes with its reproductive processes. The underlying mechanisms may involve multiple aspects, including but not limited to hormone signaling, cell division and expansion, nutrient allocation, and environmental adaptability.

**Figure 11:**
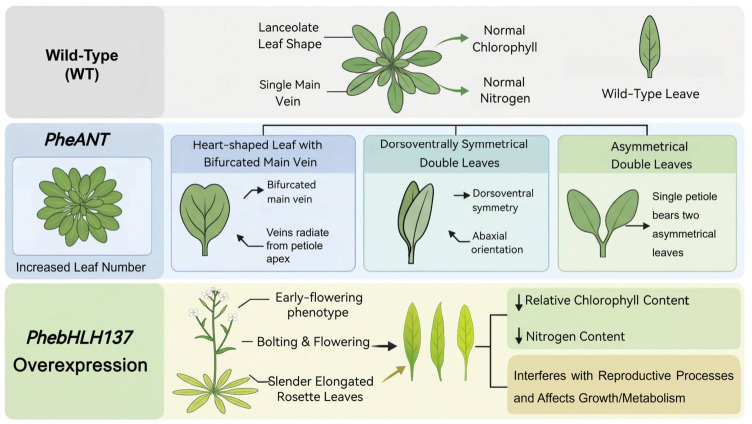
Differential regulation of leaf morphogenesis and reproductive transition in *Arabidopsis* by the Moso bamboo genes *PheANT* and *PhebHLH137*.

According to the above phenotypic analysis, the *PheANT* and *PhebHLH137* genes play distinct but critical roles in the growth and development of *Arabidopsis* (Figure 11). They exhibit significant functional differentiation: *PheANT* functions in the regulation of leaf structure during vegetative growth. By interfering with leaf primordium polarity establishment and vascular patterning, it induces aberrant phenotypes such as cordate leaves, forked midveins, and dorsiventral symmetric/asymmetric double leaves, while also significantly increasing leaf number per plant. This suggests that *PheANT* integrates pathways controlling leaf number and leaf shape determination, thereby remodeling leaf architecture. In contrast, *PhebHLH137* exerts a metabolic regulatory function during the transition to reproduction. Overexpression of this gene leads to early bolting, elongated rosette leaves, and a concurrent decrease in chlorophyll and nitrogen contents, indicating that it may accelerate the shift from vegetative to reproductive growth by upregulating floral integrators, while simultaneously suppressing genes related to photosynthesis and nitrogen assimilation, thereby prioritizing resources for reproductive growth. Together, these two genes reveal the molecular regulatory logic governing the vegetative and reproductive phases during plant development, from the perspectives of morphogenesis and metabolic remodeling, respectively.

## 4. Discussion

### 4.1. Sequencing Data Quality Assessment and Differential Gene Screening Strategy

In this study, high-quality transcriptome data were obtained via Illumina RNA sequencing. The proportion of clean reads in each sample exceeded 99%, and the mapping rate to the reference genome reached as high as 94.84–96.12%, indicating that the sequencing data were reliable and well-matched to the Moso bamboo reference genome, thereby establishing a solid foundation for subsequent differential expression analysis. Principal component analysis revealed clear separation of samples at different developmental stages of Moso bamboo leaves in the principal component space, with extremely high correlation among biological replicates, while the correlation among samples from different stages decreased as development progressed, suggesting a systematic transcriptome reprogramming during leaf development. On this basis, a stringent threshold of FDR < 0.05 and |log2FC| > 2 was adopted to identify differentially expressed genes. As shown in the volcano plot (Figure 2c), the vast majority of differentially expressed genes had |log2FC| values significantly greater than 2, with upregulated and down-regulated genes clearly separated from non-differentially expressed genes, and very few genes fell near the threshold line. This indicates that key genes involved in Moso bamboo leaf development generally exhibit large-magnitude expression changes, while genes with small changes are limited in number and often fail to meet the FDR < 0.05 criterion. Therefore, this threshold helps filter out technical noise and obtain a high-confidence set of differentially expressed genes. At the same time, we acknowledge that a stringent threshold may miss some functionally relevant genes with smaller expression changes. Using the above criteria, a total of 25,256 differentially expressed genes were identified, among which 3426 were shared across the three comparison groups. This suggests a cumulative effect of gene expression changes from the division stage to the elongation stage and then to the maturation stage. Moreover, the extensive overlap of differentially expressed genes between the maturation stage and the two early developmental stages further supports the existence of a gradually established functionally mature transcriptome program during leaf development.

### 4.2. Multi-Level Gene Regulatory Network of Leaf Development in Moso Bamboo

Leaf development in Moso bamboo is a morphogenetic process precisely controlled by a multi-level gene regulatory network. Through comparative transcriptome analysis, this study systematically revealed the patterns of molecular events from leaf primordium initiation to functional maturation, and discusses them from the perspectives of developmental stage specificity, hormonal regulation, carbon metabolism, photosynthetic system establishment, and transcription factor coordination. Our results delineate the stage-specific molecular characteristics of Moso bamboo leaf development, which are consistent with the general principles of plant leaf development [25]. Meanwhile, KEGG pathway enrichment analysis identified plant hormone signal transduction and various sugar metabolism pathways (Figure 4a), suggesting that plant hormones and energy play central roles in cell division and expansion during early leaf development [26]. As leaves progressively developed toward maturity, the enrichment profile shifted toward photosynthesis and flavonoid biosynthesis, marking the establishment of photosynthetic function and the accumulation of secondary metabolites.

Plant hormones play a central regulatory role throughout all stages of leaf development, with different hormonal pathways exhibiting dynamic synergistic and antagonistic relationships [27]. During the early stages of leaf development, the auxin and cytokinin pathways are preferentially activated. In the auxin pathway, while *TIR1* and *ARF* are downregulated, the AUX/IAA family, which serves as transcriptional repressors of auxin signaling, exhibits differential expression [28]. This differential expression pattern of AUX/IAA aligns closely with the role of auxin in precisely controlling cell proliferation and polarity establishment during leaf primordium initiation [29]. In the cytokinin pathway, the early upregulation followed by downregulation of *CRE1* is consistent with its function in promoting cell division. Meanwhile, Type-A ARRs, acting as negative feedback inhibitors in this pathway, are highly expressed early on and subsequently downregulated, thereby initiating a precise transition from proliferation to differentiation by timely terminating cell division [25]. In the GA pathway, the early high expression of *GID1* promotes cell proliferation and elongation, driving the transition of primordia into mature leaves [30,31]. Furthermore, the *BKI1* membrane-bound inhibitor in the BR pathway, EBF1/2 in the ethylene pathway, and the JAZ protein family in the JA pathway also play refined inhibitory regulatory roles during leaf development [32,33,34]. Through a spatiotemporally precise network of synergy and antagonism, multiple hormones dynamically regulate cell proliferation thresholds, polarity establishment, maturation, and senescence, thereby ensuring normal leaf development.

Carbohydrate metabolism supplies energy and structural materials for leaf development [35]. During early development, the high expression of PGI and PFK in the glycolytic pathway accelerates ATP production, providing immediate energy for cell division and expansion [36]. Meanwhile, the starch and sucrose metabolic pathways are active, supplying raw materials for cell wall synthesis [37,38]. In later stages, the upregulated expression of AGP and PGM promotes starch synthesis in mature leaves as a carbon reserve [39], while the downregulation of GBSS and SSS reduces starch degradation to minimize energy consumption [40]. The downregulation of OGDH in the TCA cycle indicates a shift in energy demand patterns [41]. Overall, carbohydrate metabolism exhibits an upward trend, reflecting a metabolic transition from relying on catabolic energy production in early development to integrating photosynthesis and carbon reserve accumulation in later stages, which is consistent with the functional shift of the leaf from a “sink” to a “source” [25].

The establishment of photosynthesis is a core hallmark of leaf functional maturation. The differential expression of 75 photosynthesis-related genes revealed the progressive assembly process of the photosynthetic apparatus in Moso bamboo leaves. The significant upregulation of the light-harvesting complex proteins Lhca and Lhcb represents the first step in initiating the photosynthetic system, enhancing light energy capture efficiency, which is consistent with the conclusion that *Lhca1* expression is associated with photosynthetic function in *Pseudosasa japonica* [42]. The coordinated upregulation of genes encoding core components of photosystem I (PSI) and photosystem II (PSII), the cytochrome b6/f complex, and genes involved in the photosynthetic electron transport chain collectively promotes the assembly of the reaction center, enhances electron transport efficiency, and facilitates the generation of ATP and NADPH [43,44,45]. This pattern of coordinated upregulation delineates a dynamic process from the construction to the efficient operation of the photosynthetic system, laying the material and energy foundation for the rapid growth of Moso bamboo.

Transcription factors, acting as upstream regulatory hubs, play critical roles in leaf morphogenesis [28,46]. In this study, 15 core transcription factors were screened from 56 families, with functions involving the regulation of leaf shape, polarity, and size. Among these, the ARR-B and bHLH families regulate leaf shape; the ARF, HB, and C2C2-YABBY families coordinately regulate leaf polarity (the conserved function of YABBY in abaxial fate determination has been demonstrated [46]); and the GRF and AP2-EREBP families regulate leaf size, consistent with their roles in promoting cell proliferation [25]. These transcription factors form a complex regulatory network that collectively controls leaf morphogenesis in Moso bamboo.

### 4.3. Functional Analysis of PheANT in Regulating Leaf Number and Morphogenesis

In this study, heterologous expression of *PheANT*, a member of the AP2/EREBP gene family from Moso bamboo, revealed that this nucleus-localized transcription factor, when overexpressed in *Arabidopsis*, induced striking leaf morphological alterations. Among the observed abnormalities, the most frequent was the differentiation of two asymmetrical leaves at a single petiole tip, accompanied by disrupted leaf venation and the emergence of bifurcated main veins radiating from the petiole tip (Figure 9b–e). These phenotypes suggest that *PheANT* may be involved in the regulation of leaf initiation, vein patterning, and leaf polarity establishment, which is consistent with the known function of AP2/EREBP transcription factors in simultaneously regulating leaf shape and size during leaf development [47]. In 84K poplar, AP2/EREBP family members exhibit dynamic expression patterns at different stages of leaf development, implying their critical roles in leaf morphogenesis [47]. The increased leaf number and abnormal differentiation of the petiole apical meristem observed in this study are closely related to the function of the *AINTEGUMENTA* (*ANT*) gene in maintaining shoot apical meristem activity. Previous studies have shown that *ANT* and its homologs *AIL6* and *AIL7* play essential roles in maintaining the shoot apical meristem in *Arabidopsis*; loss of function of these three genes results in plants producing only a few aberrant leaves [48]. *ANT*, *AIL6*, and *AIL7* maintain meristem indeterminacy by regulating cell division in the meristematic zone and the expression of key regulators such as *WUSCHEL* (*WUS*), *CLAVATA3* (*CLV3*), and *SHOOT MERISTEMLESS* (*STM*) [48]. The increased leaf number upon *PheANT* overexpression in this study may therefore be attributed to enhanced leaf primordium initiation frequency or sustained meristematic activity promoted by this gene. The disturbed leaf venation and bifurcated main vein phenotype resulting from *PheANT* overexpression echo the regulatory function of maize (*Zea mays*) *ANT1* (*ZmANT1*) in vascular development. It has been shown that *ZmANT1* participates in the regulation of vascular and chloroplast development by binding to the promoters of key regulators such as *SCR1*, *GNC*, and *AN3*, and mutations in *ZmANT1* lead to aberrant vascular structures and disrupted vein spacing [49]. The bifurcated vein phenotype caused by *PheANT* overexpression in this study suggests that this gene may be involved in the regulatory network of vascular patterning, and its ectopic expression likely interferes with the spatiotemporal precision of vein differentiation signals. Regarding leaf polarity establishment, the observed phenotypes of dorsoventrally symmetrical double leaves and the differentiation of two leaves at a single petiole tip indicate that *PheANT* may influence the regulatory pathways controlling leaf polarity determination. Similar phenomena have been reported for other members of the AP2/EREBP family: ectopic expression of *DoEREB5* from *Dendrobium officinale* in *Arabidopsis* also altered leaf morphology and petiole size [50], and overexpression of SHINE (SHN) transcription factors not only altered leaf epidermal cell structure, trichome number, and branching pattern but also affected stomatal index [51]. Collectively, these studies demonstrate that AP2/EREBP family members act at multiple levels of leaf morphogenesis by regulating cell proliferation, differentiation, and polarity establishment. Considering the known mechanism of *ANT* in regulating cell proliferation–controlling organ size by promoting cell division and prolonging the period of cell proliferation [52]–*PheANT* may similarly regulate the expression of cell cycle-related genes. The diverse leaf morphological phenotypes observed in this study likely result from the perturbation by *PheANT* overexpression of the spatiotemporal coordination of multiple regulatory networks, including cell proliferation, differentiation, vascular patterning, and polarity determination during leaf development.

### 4.4. Mechanisms Underlying the Pleiotropic Developmental Phenotypes in Arabidopsis Caused by PhebHLH137 Overexpression

Heterologous expression of Moso bamboo *PhebHLH137* induced pleiotropic developmental phenotypes in *Arabidopsis*. Transgenic *Arabidopsis* overexpressing *PhebHLH137* exhibited an early-flowering phenotype (Figure 10), indicating that this gene may be involved in flowering activation, which is consistent with the known function of bHLH transcription factors in flowering regulation [53,54]. Overexpression of *PhebHLH137*, a Moso bamboo homolog, in *Arabidopsis* similarly promoted flowering, suggesting that it may act on a conserved flowering regulatory node [20]. This early flowering phenotype suggests that *PhebHLH137* may be involved in regulating the flowering transition; however, its precise molecular mechanism and regulatory effects on key flowering genes remain to be further investigated. The transgenic plants showed slender, elongated rosette leaves with decreased leaf width, increased length-to-width ratio, and elongated petioles. Meanwhile, the relative chlorophyll content and nitrogen content were significantly reduced, and the leaves appeared yellowish (Figure 10f,g), indicating that *PhebHLH137* may participate in leaf morphogenesis and the regulation of photosynthetic pigment metabolism. Additionally, this might be related to the role of *bHLH137* in regulating anthocyanin accumulation [55,56]. The *Arabidopsis bhlh93* mutant exhibits curled, dark green leaves, in contrast to the overexpression phenotype observed in this study, suggesting that the two genes may have opposing functions or act on different downstream pathways [57]. The coordinated reduction in chlorophyll and nitrogen content suggests that *PhebHLH137* may interfere with the coupling between nitrogen metabolism and photosynthesis. Subcellular localization and expression analyses confirmed that *PhebHLH137* is localized in the nucleus and highly expressed in transgenic plants, supporting its role as a transcription factor regulating downstream genes. Given the pleiotropic phenotypes, *PhebHLH137* likely regulates gene expression across multiple developmental pathways. Referring to the mechanism by which *bHLH93* promotes flowering by repressing *MAF5* [57], *PhebHLH137* may similarly regulate flowering repressors or GA metabolism genes. The leaf and fertility phenotypes suggest that its downstream target genes may involve those related to the cell cycle, chloroplast development, or floral organ identity determination. In summary, *PheANT* and *PhebHLH137* represent the key regulatory roles of the two major transcription factor families, AP2/EREBP and bHLH, respectively, in Moso bamboo leaf development. *PheANT* primarily affects leaf initiation, vascular patterning, and polarity establishment, whereas *PhebHLH137* is involved in flowering transition, leaf morphology, and photosynthetic pigment metabolism. Together with the hormone signaling, carbon metabolism, and photosynthetic system establishment within the aforementioned integrated network, these two genes collectively constitute a multi-level, multi-factor synergistic regulatory system governing leaf development in Moso bamboo.

In conclusion, leaf development in Moso bamboo is a dynamic process synergistically regulated by multiple genes and pathways: the early stage is characterized by hormone signaling initiation and polarity establishment, accompanied by energy metabolism; the middle stage shifts toward active biosynthesis and ribosome biogenesis; and the later stage focuses on the assembly and optimization of the photosynthetic apparatus as well as the initiation of secondary metabolism. Plant hormones, carbohydrate metabolism, photosynthesis-related genes, and core transcription factors collectively weave a complex regulatory network that ensures the coordination of leaf morphology and function. On this basis, through heterologous expression, this study further revealed the specific functions of two key transcription factors: *PheANT* affects leaf number, vein pattern, and leaf shape by regulating cell proliferation, meristem activity, vascular patterning, and polarity establishment; *PhebHLH137* is involved in flowering transition, leaf morphogenesis, and photosynthetic pigment metabolism, and its overexpression leads to phenotypes such as early flowering, narrow and elongated leaves, and reduced chlorophyll content. These two types of transcription factors intertwine with hormone signaling, carbon metabolism, and photosynthetic system establishment within the overall developmental network, collectively forming a multi-level, multi-factor synergistic regulatory system governing leaf development in Moso bamboo. This study deepens our understanding of the molecular mechanisms underlying leaf development in bamboo plants and provides important genetic resources and theoretical foundations for the genetic improvement and efficient cultivation of Moso bamboo.

## Data Availability

The raw sequence datasets from this research have been submitted to the NGDC GSA database under the project numbers: CRA042279. The data are publicly accessible at the following URL: https://ngdc.cncb.ac.cn/gsa (accessed on 29 April 2026).

## Supplementary Materials

The following supporting information can be downloaded at: https://www.mdpi.com/article/10.3390/plants15111673/s1. Figure S1. GO functional enrichment analysis of differentially expressed genes. Note: Bar charts showing the GO secondary classification of differentially expressed genes in pairwise comparisons, illustrating the distribution of gene counts across subcategories within the three major domains: Biological Process (BP), Molecular Function (MF), and Cellular Component (CC). Red represents up-regulated genes, and blue represents down-regulated genes; Table S1. Information table of primers; Table S2. Summary of Illumina RNA sequencing data.
