# Supplementary material for: Identification and Functional Validation of Key Regulatory Genes for Leaf Development in Moso Bamboo (Phyllostachys edulis) Based on Transcriptome Data
Source: Plants (Basel). 2026 May 29;15(11):1673. doi: 10.3390/plants15111673 (PMC13259442; doi:10.3390/plants15111673)
Supplement: Supplementary file 1 [file plants-15-01673-s001.zip › plants-4321644-supplementary.pdf]

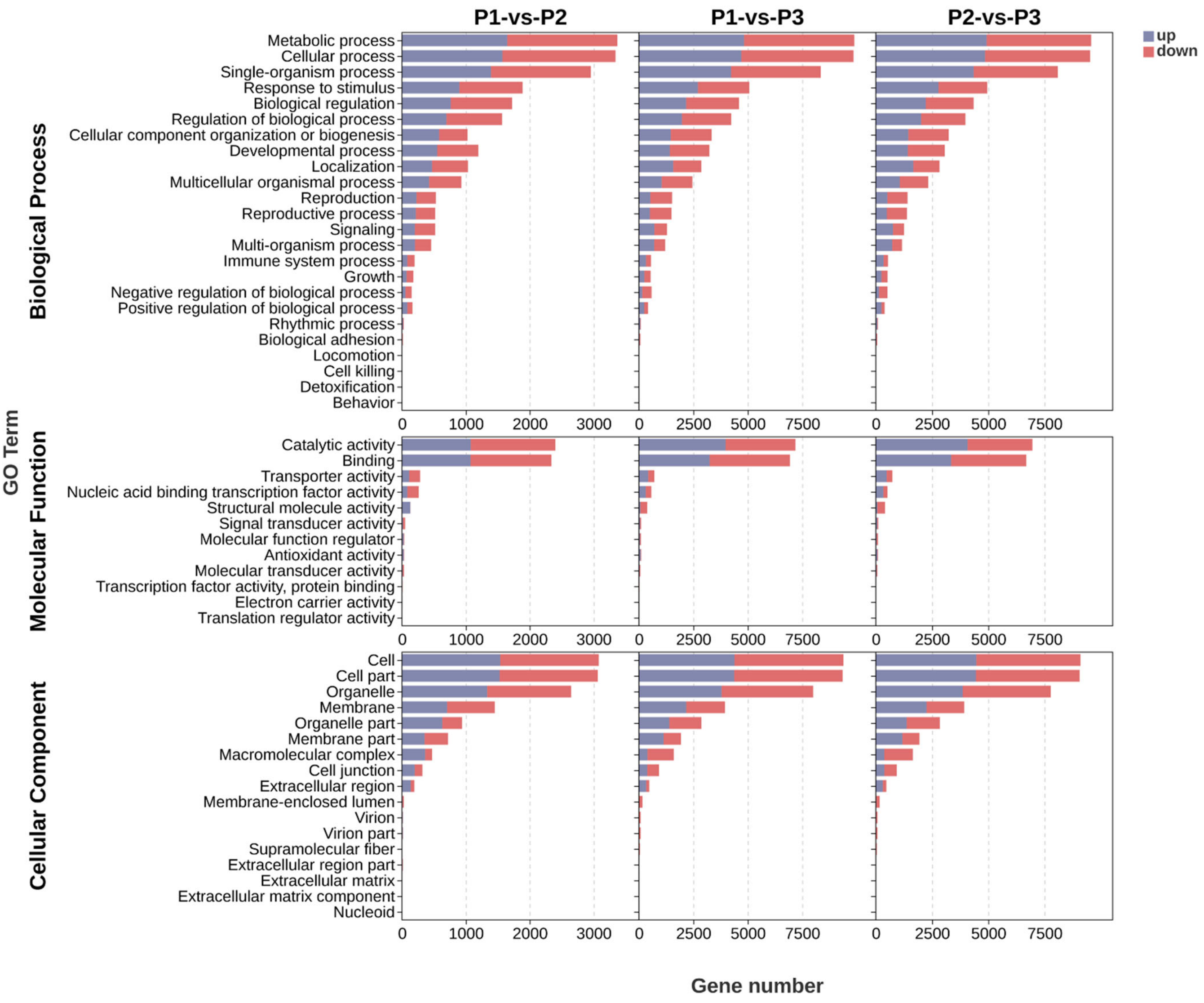

Figure S1. O functional enrichment analysis of differentially expressed genes. Note: Bar charts showing the GO secondary classification of differentially expressed genes in pairwise comparisons, illustrating the distribution of gene counts across subcategories within the three major domains: Biological Process (BP), Molecular Function (MF), and Cellular Component (CC). Red represents up-regulated genes, and blue represents down-regulated genes.

Table S1. Information table of primers

Gene name / Vector		Homology Arm	Primer Sequence
PheBHLH137	F	aacacgggggactcttgacc	ATGGCAGACTTCTCATCCCA
	R	ggaaattcgagctggtcacc	CTAGAAAGAGTACATGTTGTTGAACAC
PheANT	F	aacacgggggactcttgacc	ATGAGAGCAATGGCTAGTAGCG
	R	ggaaattcgagctggtcacc	TTATGCATCCGTCCAAGCAG
qRT-PheBHLH137	F	/	AGGATGAGGATGCTGCAAGC
	R	/	TGGTCATGGAGGCCATCACT
qRT-PhePHL7	F	/	CAAGCTCGGCAACAGATGGT
	R	/	CCTGTTGGGCTAGCCTCCTT
SD-PheBHLH137	F	aacacgggggactcttgacc	ATGGCAGACTTCTCATCCCA
	R	agttcttctcctttactagt	GAAAGAGTACATGTTGTTGAACACC
SD- PhePHL7	F	aacacgggggactcttgacc	ATGAGAGCAATGGCTAGTAGCG
	R	agttcttctcctttactagt	TGCATCCGTCCAAGCAGC
pCAMBIA1302	F	/	GAACACGGGGGACTCTTGAC
	R	/	CAAGACCGGCAACAGGATTC
At-actin	F	/	CTCTCCGCTTTGAATTGTCTCGTTG
	R	/	GGTACCATTGTCACACACGATTGGT

Table S2. Summary of Illumina RNA sequencing data

Sample	RawDatas	CleanData (%)	Total reads	Total_Mapped (%)
P1-1	44139542	44011666 (99.71%)	43983410	42094980 (95.71%)
P1-2	42580302	42361890 (99.49%)	42332282	40514449 (95.71%)
P1-3	48373984	48035236 (99.30%)	48002538	45523985 (94.84%)
P2-1	43703804	43580586 (99.72%)	43533358	41843135 (96.12%)
P2-2	44809958	44582192 (99.49%)	44532982	42765898 (96.03%)
P2-3	47280816	46963440 (99.33%)	46912624	44645930 (95.17%)
P3-1	42718764	42591634 (99.70%)	42533570	40779984 (95.88%)
P3-2	45084192	44919786 (99.64%)	44848608	43019050 (95.92%)
P3-3	45751062	45606364 (99.68%)	45532270	43660293 (95.89%)
